# Circulating adiponectin and leptin and risk of overall and aggressive prostate cancer: a systematic review and meta-analysis

**DOI:** 10.1038/s41598-020-79345-4

**Published:** 2021-01-11

**Authors:** Anya J. Burton, Rebecca Gilbert, Kate Tilling, Ryan Langdon, Jenny L. Donovan, Jeff M. P. Holly, Richard M. Martin

**Affiliations:** 1 Bristol Medical School, Translational Health Sciences, University of Bristol, Learning and Research Building, Level 2, Southmead Hospital, Bristol, UK; 2 Bristol Medical School, Population Health Sciences, University of Bristol, Bristol, UK; 3 National Institute for Health Research (NIHR) Bristol Biomedical Research Centre at University Hospitals Bristol and Weston NHS Foundation Trust and the University of Bristol, Bristol, UK

**Keywords:** Cancer, Epidemiology

## Abstract

Obesity is associated with an increased risk of advanced, recurrent and fatal prostate cancer. Adipokines may mediate this relationship. We conducted a systematic review and meta-analysis of associations of leptin and adiponectin with overall and aggressive prostate cancer. Bibliographic databases were **s**ystematically searched up to 1st April 2017. Log Odds Ratios (ORs) per 2.5 unit increase in adiponectin or leptin levels were derived and pooled. All analyses were stratified by study type (cross-sectional/prospective). 746 papers were retrieved, 34 eligible studies identified, 31 of these could be included in the meta-analysis. Leptin was not consistently associated with overall prostate cancer (pooled OR 1.00, 95%CI 0.98–1.02, per 2.5 ng/ml increase, prospective study OR 0.97, 95%CI 0.95–0.99, cross-sectional study OR 1.19, 95%CI 1.13–1.26) and there was weak evidence of a positive association with aggressive disease (OR 1.03, 95%CI 1.00–1.06). There was also weak evidence of a small inverse association of adiponectin with overall prostate cancer (OR 0.96, 95%CI 0.93–0.99, per 2.5 µg/ml increase), but less evidence of an association with aggressive disease (OR 0.98, 95%CI 0.94–1.01). The magnitude of any effects are small, therefore levels of circulating adiponectin or leptin alone are unlikely to be useful biomarkers of risk or prognosis.

## Introduction

Prostate cancer is a cause of considerable morbidity and mortality, particularly in industrialised countries where obesity is epidemic^1,2^. Although over 60% of men aged 85 have histological evidence of prostate cancer^3^, the disease is often indolent and most affected men will die of other causes before the cancer progresses^4^. Identification of markers of aggressive disease is imperative to recognising those cancers likely to progress, enabling radical treatment to be reserved for high-risk cases and minimising morbidity from unnecessary treatments^5,6^. In meta-analyses of observational studies, body mass index (BMI) is associated with a modest increased risk of advanced and fatal prostate cancer, and of biochemical recurrence (8–21% increase in risk per 5 kg/m^2^ increment in BMI)^7–9^. However, Mendelian randomisation analysis did not find genetic variants associated with increased BMI to be associated with risk of advanced or high grade disease but did find weak evidence of an association with lower prostate cancer risk^10^.

Biologically-active polypeptides synthesised and secreted by white adipose tissue, adipokines, may mediate the association between obesity and prostate cancer progression. In vitro, leptin stimulates growth factor expression^11^, proliferation^12^, androgen-independent cells migration^13^ and expresses angiogenic properties^14^, while adiponectin inhibits prostate cancer cell proliferation^15^ and angiogenesis^16^. We hypothesise that leptin is positively associated with risk of overall and aggressive prostate cancer and adiponectin is inversely associated with risk of overall and aggressive prostate cancer. Here we systematically review the epidemiological evidence on associations of circulating adiponectin and leptin with overall and aggressive (higher grade and/or more advanced stage) prostate cancer and combine study-specific effect estimates in a dose–response meta-analysis.

## Methods

### Search strategy

Studies in humans of associations of circulating adiponectin and/or leptin with prostate cancer prevalence, incidence, stage, grade, mortality or other measures of aggressive prostate cancer were identified through systematic searches of the bibliographic databases Medline (1950-April 1st 2017), Embase (1980-April 1st 2017) and Web of Science (1899-April 1st 2017). A comprehensive combination of MeSH and text words was used to search the databases (Supplementary Table S1) and no language restrictions were applied. Reference lists of the articles selected for inclusion in the meta-analysis and of related articles, particularly reviews, were searched by hand.

### Inclusion and exclusion

Abstracts were screened and excluded if they were: genetic studies, animal studies, cell culture or biochemical studies, if they did not report on associations of adiponectin or leptin with prostate cancer, or if they did not contain original data (i.e. were review papers and/or commentaries). For the remaining studies and those for which eligibility was unclear, full articles were retrieved and assessed for inclusion by two reviewers independently (AB, and KT, RMM or RL). Studies were eligible for inclusion if they: presented original peer-reviewed data, included measures of adiponectin and/or leptin in human blood, included data from men with prostate cancer and included a comparison group (for overall prostate cancer incidence this was cancer-free men and for aggressive prostate cancer incidence this was either men without cancer or with low risk prostate cancer). Cohort, nested case–control and retrospective case–control studies were eligible. Duplicate publication of study results was identified by comparing study locations, authors, study names, descriptions of the study population, recruitment dates and study designs. Where results were published more than once, the paper which included the highest number of cases, the most detail or the most comparable estimate was selected for inclusion.

### Data extraction

Data at the level of the study (e.g. year of publication, author, study type) and the result (e.g. estimate type, number of cases and controls, covariates included) were extracted using a standardised extraction form by one author (AB or RL) and check by another (RB). If data necessary to derive a dose–response odds ratio (OR) were not extractable (risk estimate not given, risk estimate scale not given, or the distribution of adiponectin or leptin levels not given), study authors were contacted for further details.

### Statistical analysis

To combine data across studies, study-specific estimates were converted to log OR per 2.5 unit (ng/ml for leptin and µg/ml for adiponectin) increase in adiponectin or leptin. These ‘dose–response’ ORs were pooled in a meta-analysis, separately for overall prostate cancer risk and risk of aggressive disease (defined as high grade, advanced stage, high volume, a combination measure and/or fatal prostate cancer). A 2.5 unit increase was selected as this represents approximately one quarter of the adiponectin or leptin distribution and a 2.5 ng/ml increase in leptin corresponds to around a 5 kg/m^2^ increase in BMI (calculated from the distribution in the Prostate Testing for Cancer and Treatment study^17^. The limited number of studies identified prevented the possibility of assessment of non-linear associations.

Results were reported in three main forms, each of which required a different method of conversion. Firstly, if the difference in means or medians between cases and controls was reported, this was converted to dose–response ORs using the method described by Chêne and Thompson, which assumes an approximately normal distribution of exposure^18^. Secondly, if the ORs per quantile of adiponectin or leptin were reported, a dose–response OR was derived using the Greenland and Longnecker method^19^. For this method, a mean or median of the exposure in each quantile and the number of cases and controls in each quantile, were needed; if neither of these were reported, the mean in each group was estimated using the range^18^. Thirdly, if the odds ratio per (x) units increase in exposure was given, the OR per 1 unit was calculated by 1 − ((1 − OR)/x). Following conversion to a log odds ratio, this was then multiplied by 2.5 to give the log odds ratio per 2.5 units.

Two primary meta-analyses were carried out for each exposure: (i) a pooled estimate of the log OR of overall prostate cancer risk per 2.5 unit increase in adiponectin or leptin; and (ii) a pooled estimate of the log odds ratio of aggressive prostate cancer per 2.5 unit increase in adiponectin or leptin. Analyses were stratified by study design; if blood draw was at any time point before biopsy this was considered prospective (including diagnostic PSA tests, 5 studies), and if after biopsy, as cross-sectional. If a paper presented more than one type of result, or more than one logistic regression result (i.e. minimally adjusted and multivariable adjusted), all results were extracted but the results were selected for inclusion in the main analyses using the following order of priority: (A) the effect estimate was: (i) a dose–response OR, (ii) a categorical/quantile OR, (iii) the median or mean difference between cases and controls; (B) the estimate was adjusted for: (i) age only (minimally adjusted as these were more comparable between studies), (ii) hormones/smoking/BMI (maximally adjusted); (C) the measure of aggressiveness was: (i) grade (most commonly reported and therefore was most comparable between studies); (ii) stage; (iii) another measure (such as high volume disease, mortality or a combination measure).

As weights applied in fixed-effects analyses are more proportional to the size of the study, the primary analyses were based on the fixed effects estimates, although random effect estimates (which generally give more conservative estimates than fixed effects estimates but give more weight to smaller studies^20^) were also calculated and presented for completeness. The I^2^ statistic was calculated to quantify the percentage of between-study variation due to heterogeneity^21,22^; an I^2^ of 0% indicates the true association is the same between studies. The larger the I^2^ the higher the proportion of the total variation in study estimates is due to between-study variability and not sampling error, indicating that the true association differs between studies^22^.

Subgroup analyses of the main results were used to explore potential sources of heterogeneity: (i) the original results format used to derive the dose–response OR (mean difference or odds ratio)); (ii) the assay method (ELISA, RIA or another)); (iii) the measure of prostate cancer aggressiveness (grade, stage, other/combined); (iv) the mean study-level BMI (< or ≥ 27kgm^2^) ; and v) the method of prostate cancer detection i.e. whether non-PSA screen (clinically) detected or detected by PSA-screening. If studies did not specifically report whether cases were PSA or clinically detected, they were classified as method ‘not reported’, apart from those with high mean PSA levels amongst cases or those where cases were sampled before the PSA screening era, which were classified as non-PSA screen detected. Where reported, we extracted effect-estimates stratified by BMI (usually < 25 and ≥ 25kgm^2^). We also assessed heterogeneity by factors that could affect susceptibility to bias, as there is not a single generally accepted quality assessment tool for observational studies^23^: study design (prospective versus cross-sectional data collection, as defined above); adjustment for confounding (including maximally adjusted models over minimally adjusted models); and type of effect-estimate (comparing pooled OR estimates to pooled mean difference estimates).

### Sensitivity analysis and publication bias

We conducted sensitivity analyses where: (i) advanced stage was selected over other measures of aggressive disease and over high grade; (ii) maximally adjusted estimates were selected over minimally adjusted and (iii) both (i) and (ii). Some prospective studies drew blood around the time of diagnosis, at which point the cancer would have already been present which may affect adiponectin or leptin levels (reverse causality). To examine the effect of time of blood draw relative to diagnosis prospective studies were plotted by time of blood draw and an additional sensitivity analysis redefining prospective as > 1 year pre-diagnosis was conducted. An influence analysis, in which each study was excluded from the pooled estimate systematically, was conducted to gauge the influence of individual studies on pooled estimates. Small study effects were explored using funnel plots^24^ and tests of funnel plot asymmetry (the Egger and the Begg tests)^25^; as these tests can produce false-positive results when analysing odds ratios^25^, manual inspection of funnel plots was used as the main indicator of publication bias.

The search was updated to October 2018 to identify if any further studies had been published after the end of the study period. These further studies, plus those from the earlier search which could not be included in the main analyses, had minimal statistical information (the P value, sample size and direction of effect) extracted and plotted alongside the main results in an albatross plot. Albatross plots allow an approximate estimation of underlying effect sizes and can potentially identify sources of heterogeneity in results from systematic reviews where limited comparable data are available^26^.

All analyses were conducted in Stata (StataCorp. 2013. Stata Statistical Software: Release 13.1. College Station, TX: StataCorp LP). The protocol for the review was registered in the PROSPERO international prospective register of systematic reviews: (http://www.crd.york.ac.uk/PROSPERO/display_record.php?ID=CRD42017074010).

## Results

### Characteristics of included studies

**Figure 1:**
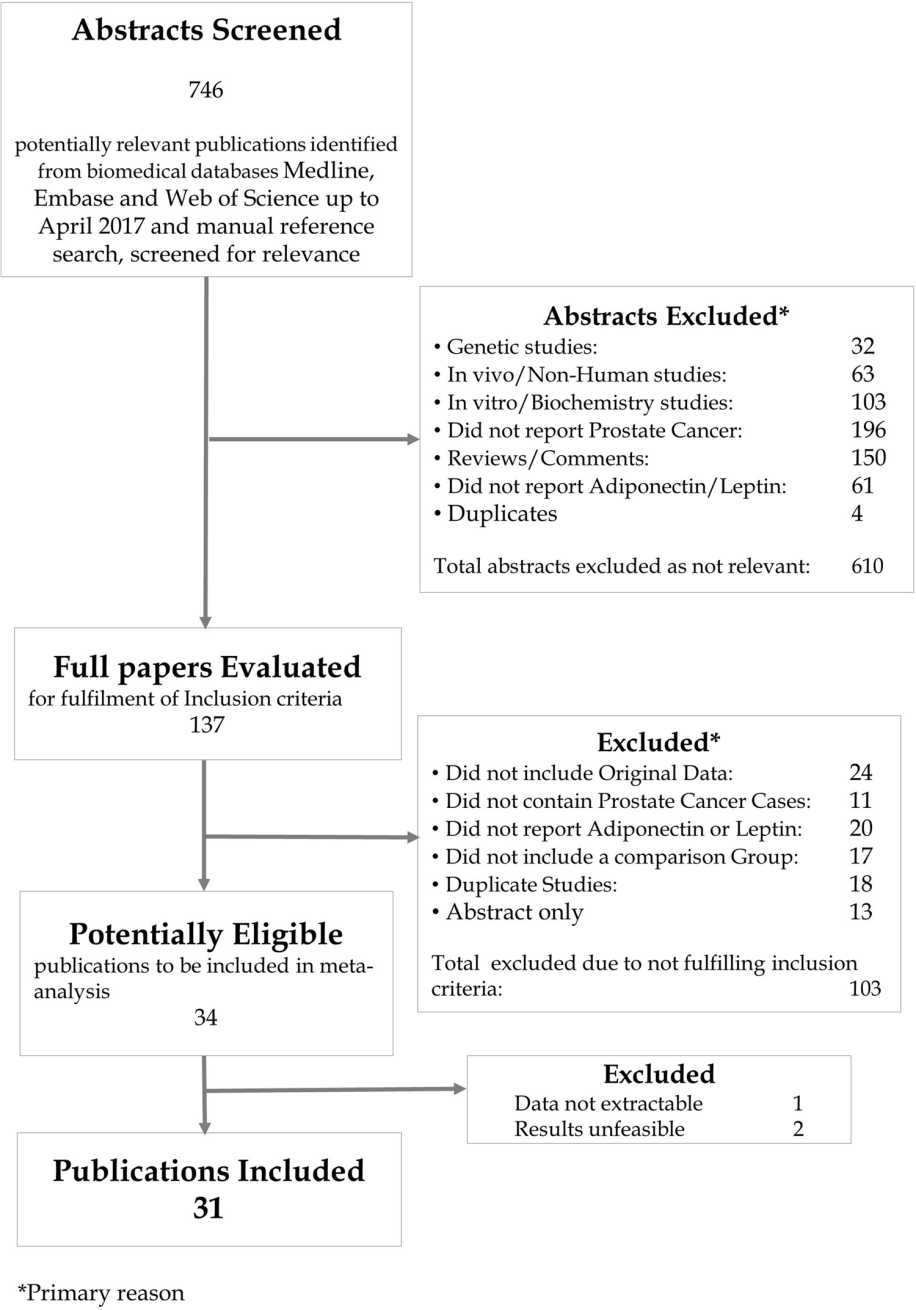
Flow diagram of study selection.

746 potentially eligible studies were identified, 610 were clearly ineligible (they were genetic, in vivo, in vitro or biochemical studies, reviews, commentaries or duplicates or did not report on the associations of adiponectin or leptin with prostate cancer) therefore 137 publications were retrieved. 34 studies fulfilled the inclusion criteria (Fig. 1). The authors of 15 of these were contacted to request further information and 9 responded and supplied additional data. Given the available data, we were able to derive dose–response ORs from all but 1 study^27^. In one small study (9 cases)^28^ the derived OR for the association between adiponectin and aggressive prostate cancer was implausibly small (0.02, 95% CI 0.00 to 0.13) and this estimate was excluded (but the effect estimate from this study for the association between adiponectin and overall prostate cancer was included). In another study it was unclear what measure of dispersion around the mean was reported and assuming either standard error or standard deviation resulted in implausibly narrow confidence intervals or small effect estimate, respectively. As it was unclear which, if either, was correct this study was excluded^29^.

Reported adipokine levels fell outside of the physiological range (3 to 30 μg/mL for adiponectin^30^ and 1 to 30 ng/ml for leptin^31^) in 7 studies of adiponectin and 1 of leptin. Adiponectin requires a dilution before analysis and it appeared five studies either failed to back-adjusted results for this dilution factor or erroneously reported results in ng/ml rather than µg/ml. When contacted, authors of two out of the five studies^32,33^ stated the former was most likely; there was no response from the remaining three studies^29,34,35^. When an adjustment for dilution factor (1:500) was made the results fell into the physiological range and these adjusted results were used in analyses. One study gave no units^36^ but when contacted the authors stated the units should be ng/ml. These results fell out of the physiological range, even after assuming this was an error and therefore this study was excluded. Another adiponectin study reported results in pg/ml, which the authors stated was a typographical error^37^. The leptin study reported results in pg/ml and when contacted the author no longer had access to the data^38^. This was assumed to be a typographical error also.

The final meta-analysis included 31 studies (Fig. 1)^17,28,32–35,37–61^, 19 studies investigated adiponectin (9 prospective, 10 cross-sectional) and 21 investigated leptin (11 prospective, 10 cross-sectional) (Table 1). The main leptin meta-analyses included 4343 incident prostate cancer cases and 1486 aggressive prostate cancer cases. The main adiponectin meta-analyses included 1550 incident prostate cancer cases and 1334 aggressive prostate cancer.

**Table 1:** Study characteristics.

First Author, Year	Prospective or cross-sectional	Country	Ethnicity	Cases*	Controls*	Time of blood draw	Exposure (assay, manufacturer)	Result type	Matching criteria, adjustments and notes*
Arisan^39^, 2007	Cross-sectional	Turkey	NS	8 men with high grade (Gleason score ≥ 8) PCa	10 with low grade (Gleason score ≤ 4) PCa	After diagnosis	A (ELISA, B-Bridge Int.), L (ELISA, DRG Diagnostics)	Mean Difference	
Baillargeon^38^, 2006	Prospective	San Antonio Center for Biomarkers of Risk of Prostate Cancer (SABOR), USA	63% non-Hispanic white, 23% Hispanic, 14% black	Total PCa: 125 men with PCa Aggressive PCa: 40 men with high grade (Gleason score ≥ 7) PCa	Total: 125 healthy men Aggressive: 85 men with low grade (Gleason score ≤ 6) PCa	Mean 1.43 (SD 1.29) years before diagnosis	A, L (MAP, Luminex)	Categorical OR (tertiles)	Adjusted for age and race. Inverse normal used to estimate distribution in cases from distribution in control. Assumed the units for leptin should be ng/ml
Basaria^40^, 2005	Cross-sectional	USA	NS	Total: 17 men with non-metastatic PCa Aggressive: 18 men with metastatic PCa treated with RP or radiotherapy	Total: 18 healthy men Aggressive: 17 men with non-metastatic PCa	After diagnosis, after treatment	L (ELISA, ALPCO Diagnostics)	Mean Difference	SDs calculated from SEs
Burton^17^, 2013	Prospective	UK	98.7% white 0.6% black 0.7% other	416 men with high grade (Gleason score ≥ 7) PCa	307 men with low grade (Gleason score ≤ 6) PCa	At screening (just before diagnosis)	A, L (ELISA, R&D Systems)	Dose Response OR (per unit increase)	Adjusted for age, study centre, assay run
Chang^41^, 2001	Cross-sectional	USA	White	150 men with high volume (> 0.5 cc) PCa or extraprostatic extension	44 men with low volume (< 0.5 cc) PCa	After diagnosis	L (ELISA, Linco)	Mean Difference	
Di Sebastiano^42^, 2016	Cross-sectional	Canada	NS	8 men with aggressive PCa (Gleason ≥ 8, PSA > 20 ng/dl or stage > T3A	9 healthy men	After diagnosis	A, L (ELISA, R&D Systems)	Mean Difference	Matched for age and BMI
Di Sebastiano^37^, 2017	Cross-sectional	Canada	NS	Total: 38 with PCa Aggressive 21 men with high grade (Gleason ≥ 7) PCa	Total: 13 cancer-free men Aggressive: 17 men with low grade (Gleason) ≤ 6	At (n = 36) or after (n = 15) diagnosis	A, L (ELISA, R&D Systems)	Mean Difference	Author supplied estimates and said units for adiponectin should be µg/ml
Fowke^43^, 2013	Prospective	Nashville Men's Health Study (NMHS), USA	88.8% of high grade cases, 88.4% of low grade cases and 89.1% of controls White	Total: 95 men with low grade (Gleason score 6) PCa Aggressive: 98 men with high grade (Gleason score ≥ 7) PCa	137 biopsy-negative men	After referal for biopsy, before diagnosis	A, L (RIA, Luminex)	Mean Difference	Matched on age. Means adjusted for age, alpha-blocker use, treatment for diabetes, prostate volume, number of cores at biopsy
Freedland^44^, 2005	Cross-sectional	USA	97% white, 2% black, 1% other	75 men with more advanced stage (T2-T3) PCa	149 men with T1c stage PCa	After diagnosis, before RP	L (ELISA, NS)	Mean Difference	
Freedland^32^, 2005	Cross-sectional	USA	97% white, 2% black, 1% other	65 men with high grade (Gleason score ≥ 7) PCa	171 men with low grade (Gleason score ≤ 6) PCa	After diagnosis, before RP	A (ELISA, NS)	Categorical OR (quartiles)	Adjusted for age. Inverse normal used to estimate distribution in cases from distribution in control. Units converted from ng/ml to µg/ml
Gade-Andavolu^45^, 2006	Cross-sectional	USA	NS	55 men with PCa	54 healthy men	After diagnosis	L (RIA, NS)	Categories (≤ 7 ng/ml, > 7–14 ng/ml, > 14.1 ng/ml)	
Goktas^28^, 2005	Cross-sectional	Turkey	NS	30 men with PCa	36 healthy men	After diagnosis, before treatment	A (RIA, Linco)	Mean Difference	SDs were calculated from the p for difference. Aggressive PCa results could not be used as OR unfeasibly high
Grosman^46^, 2010	Prospective	Argentina	NS	25 men with localised PCa	25 healthy men	Before diagnosis (during diagnostic work up)	A (RIA, Linco)	Mean Difference	Age and BMI matched. SD estimated from p for difference
Gu^47^, 2014	Cross-sectional	China	100% Chinese Han	305 men with PCa	330 healthy men	After diagnosis	A (ELISA, Abcam)	Mean Difference	Matched for age and urban/rural residence
Gu^60^, 2015	Cross-sectional	China	100% Chinese Han	100 men with biochemical recurrence after radical prostatectomy	326 men without biochemical recurrence after radical prostatectomy	At diagnosis	A (ELISA, Abcam)	Mean Difference	Unmatched. SDs calculated from range (via IQR). Men are a subset of Gu 2014
Gupta^47^, 2016	Prospective	Dallas Heart Study, USA	51% black 30% white 17% Hispanic 2% other	35 men who developed PCa	1333 men healthy at blood draw, 85 of whom later developed cancer	Mean 8.1 years (IQR 4.5, 11.7) before diagnosis	L (RIA, Linco)	Mean Difference	Cohort study, denominator includes cases. SD calculated from IQR. Estimates supplied by author
Housa^33^, 2008	Cross-sectional	Czech Republic	NS	Total: 43 men with T2 stage PCa Aggressive: 16 men with high grade (Gleason score ≥ 7) PCa	Total: 25 men with BPH Aggressive: 27 men with low grade (Gleason score ≤ 6) PCa	After diagnosis, before RP	A (RIA, Linco)	Mean difference	Units converted from ng/ml to µg/ml (author said results were not adjusted for dilution factor)
Hsing^49^, 2001	Cross-sectional	China	Chinese	128 men with PCa	304 healthy men	After diagnosis, before treatment	L (RIA, Linco)	Categorical ORs (tertiles)	Adjusted for age
Ikeda^50^, 2015	Prospective	Japan	NS	Total: 24 men with PCa Aggressive 8 men with high risk PCa (D'Amico classification)	Total: 2817 men with PSA < 4 ng/ml Aggressive: 16 men with low or intermediate risk PCa (D'Amico classification)	At screening (just before diagnosis)	A, (LTIA, Otsuka)	Mean Difference	Additional data (SDs) supplied by author
Lagiou^51^, 1998	Cross-sectional	Greece	NS	43 men with PCa	48 healthy men	After diagnosis	L (RIA, Linco)	OR per 4 ng/ml increase in leptin	Age matched. Adjusted for age, height, BMI, years of schooling, sex hormones and IGF-I
Lai^52^, 2014	Prospective	Health Professionals Follow-up Study (HPFS), USA	94.2% of cases and 92.9% of controls white	1314 men who developed PCa	1314 men who did not develop PCa	Median 5.4 years (IQR 3.1–7.7 years) before diagnosis	L (ELISA, Diagnostic Systems Laboratories)	Dose Response OR (per quartile increase)	Matched on age, PSA test pre blood draw, year, time of day and season of blood draw
Li^61^, 2010	Prospective	Physicians' Health Study, USA	NS	Total: 635 men with PCa (599 in adiponectin analysis) Aggressive: 124 men with high grade (Gleason score ≥ 8) PCa (115 in adiponectin analysis)	Total: 635 healthy men (599 in adiponectin analysis) Aggressive: 124 healthy men (115 in adiponectin analysis)	Up to 18 years before diagnosis	A, L (RIA, Linco)	Categorical OR (quintiles)	Matched on and adjusted for age and smoking status
López Fontana^34^, 2011	Cross-sectional	Argentina	NS	Total: 35 men with PCa Aggressive: 23 men with high grade (Gleason score ≥ 7) PCa	Total: 35 healthy men Aggressive: 12 men with low grade (Gleason score ≤ 6) Pca	After diagnosis	A, L (ELISA, Linco)	Mean Difference	Estimate supplied by author. Units converted from ng/ml to µg/ml
Michalakis^35^, 2007	Cross-sectional	Greece	NS	75 men with PCa	150 healthy men	After diagnosis	A (RIA, Linco)	Categorical OR (quartiles)	Adjusted for age. Units converted from ng/ml to µg/ml
Neuhouser^53^, 2010	Prospective	Prostate Cancer Prevention Trial (PCPT), USA	86.1% white, 7.1% black, 6.8% other	Total: 1224 men with low grade (Gleason ≤ 6) PCa Aggressive: 486 men with high grade (Gleason score ≥ 7) PCa	1778 healthy men	Before randomisation into trial (up to 7 years before diagnosis)	L (ELISA, Diagnostic Systems Ltd.)	Categorical OR (quartiles)	Age, PCPT arm & family history matched. Adjusted for age, race, family history of PCa, finestride or placebo, smoking, baseline insulin use
Saglam^54^, 2003	Cross-sectional	Turkey	NS	Total: 21 men with PCa Aggressive: 7 men with high grade (Gleason score ≥ 8) PCa	Total: 50 healthy men Aggressive: 9 men with low grade (Gleason score 5–7) PCa	After diagnosis, before treatment	L (RIA, Linco)	Mean Difference	SDs were calculated from the p for difference
Sher^55^, 2008	Cross-sectional	USA	94% white, 4% black, 2% other	253 men with High grade (Gleason score ≥ 7) PCa	286 men with Low grade (Gleason score ≤ 6) PCa	After diagnosis, before treatment	A (ELISA, ALPCO Diagnostics)	Categorical OR (quartiles)	
Singh^56^, 2010	Cross-Sectional	India	South Asian	Total: 30 men with PCa Aggressive: 7 men with high grade (Gleason score 7) PCa	Total: 30 healthy men Aggressive: 23 men with low grade (Gleason score ≤ 6) PCa	After diagnosis	L (ELISA, DRG Diagnostics)	Mean Difference	
Stevens^57^, 2014	Prospective	Cancer Prevention Study II Nutrition Cohort (CPS-II), USA	99.6% white	272 men with aggressive prostate cancer (≥ Gleason score 7, stage T3 or T4 at diagnosis and/or fatal PCa	272 healthy men	Up to 9 years before diagnosis	A (ELISA, not stated)	Categorical OR (quartiles)	Matched on date of birth, date of blood collection, and ethnicity. Adjusted for family history of prostate cancer, BMI, physical activity, calcium intake, energy intake
Stocks^58^, 2007	Prospective	Västerbotten Intervention Project (VIP), Sweden	NS	Total: 392 men with PCa Aggressive: 114 men with aggressive (Gleason score ≥ 8, T3-4, N1 or M1, PSA > 50 ng/ml or fatal disease) PCa	392 healthy men	Up to 19 years before diagnosis—mean 6.2 years	L (RIA, Linco)	Total PCa: OR per ng/ml increase in leptin Aggressive PCa: Categorical OR (tertiles)	Age and date of recruitment matched. Inverse normal used to estimate distribution in cases of aggressive PCa from distribution in controls
Touvier^59^, 2013	Prospective	Supplémentation en Vitamines et Minéraux Antioxydants (SU.VI.MAX), France	NS	156 men who developed PCa	312 healthy men	Up to 13 years before diagnosis	A, L (ELISA, R&D Systems)	Categorical OR (quartiles)	Matched on age, BMI and intervention group

* For estimate used in the main analyses.

*PCa* prostate cancer; *ELISA* enzyme-linked immunoassay; *RIA* radioimmunoassay; *MAP* multi-analyte profiling; *LTIA* latex particle-enhanced turbidimetric immunoassay; *A* adiponectin; *L* leptin; *NS* not stated; *RP* radical prostatectomy; *BMI* body mass index; *IGF-I* insulin-like growth factor I; *BPH* benign prostatic hyperplasia.

There were more studies from the USA (n = 13) than any other country (n = 18) and, despite a wide geographical distribution including Asia, Europe, South and North America, most participants were white (see Table 1). Studies ranged from 7 to 1314 prostate cancer cases (mean 153). The average age of the men studied was 60 to 65 years. All but two studies measured adiponectin and leptin by the conventional methods of enzyme immunoassay (ELISA) or radioimmunoassay (RIA). One study used multi-analyte processing (MAP) technology^38^ and another latex particle-enhanced turbidimetric immunoassay (LTIA)^50^.

In main analyses, cases in studies of overall prostate cancer incidence were mostly a mix of men with aggressive and non-aggressive prostate cancer although four studies included men with low grade^43,53^, non-metastatic^40^, or stage T2^33^ prostate cancer only. Controls were healthy men (17 studies), men with BPH (1 study) or a mixture of both (4 studies). Cases in studies of aggressive prostate cancer were men with high grade (Gleason score ≥ 7 or ≥ 8, 13 studies), higher stage (T2-3 or metastatic, 1 study), high volume (1 study), high risk ‘D’Amico’ classification (1 study), biochemical recurrence after prostatectomy (1 study) or a combination score (defined by either high Gleason score, advanced TNM stage, high PSA level or fatal disease, 3 studies). Controls in studies of aggressive prostate cancer were men with prostate cancer who did not fall into the above categories (i.e. Gleason ≤ 6 or ‘non-aggressive’), except for 5 studies in which controls were healthy men.

### Leptin

**Figure 2:**
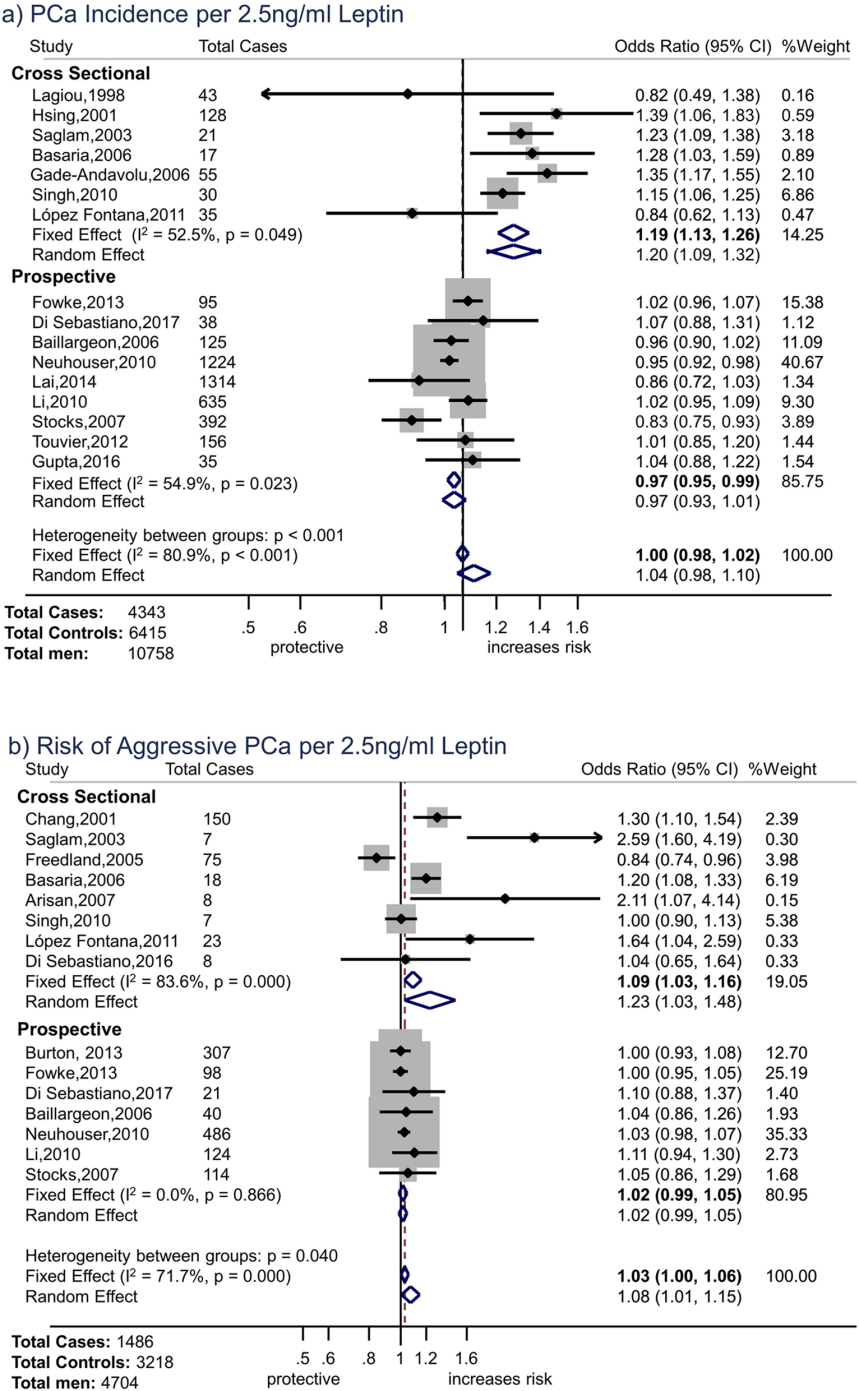

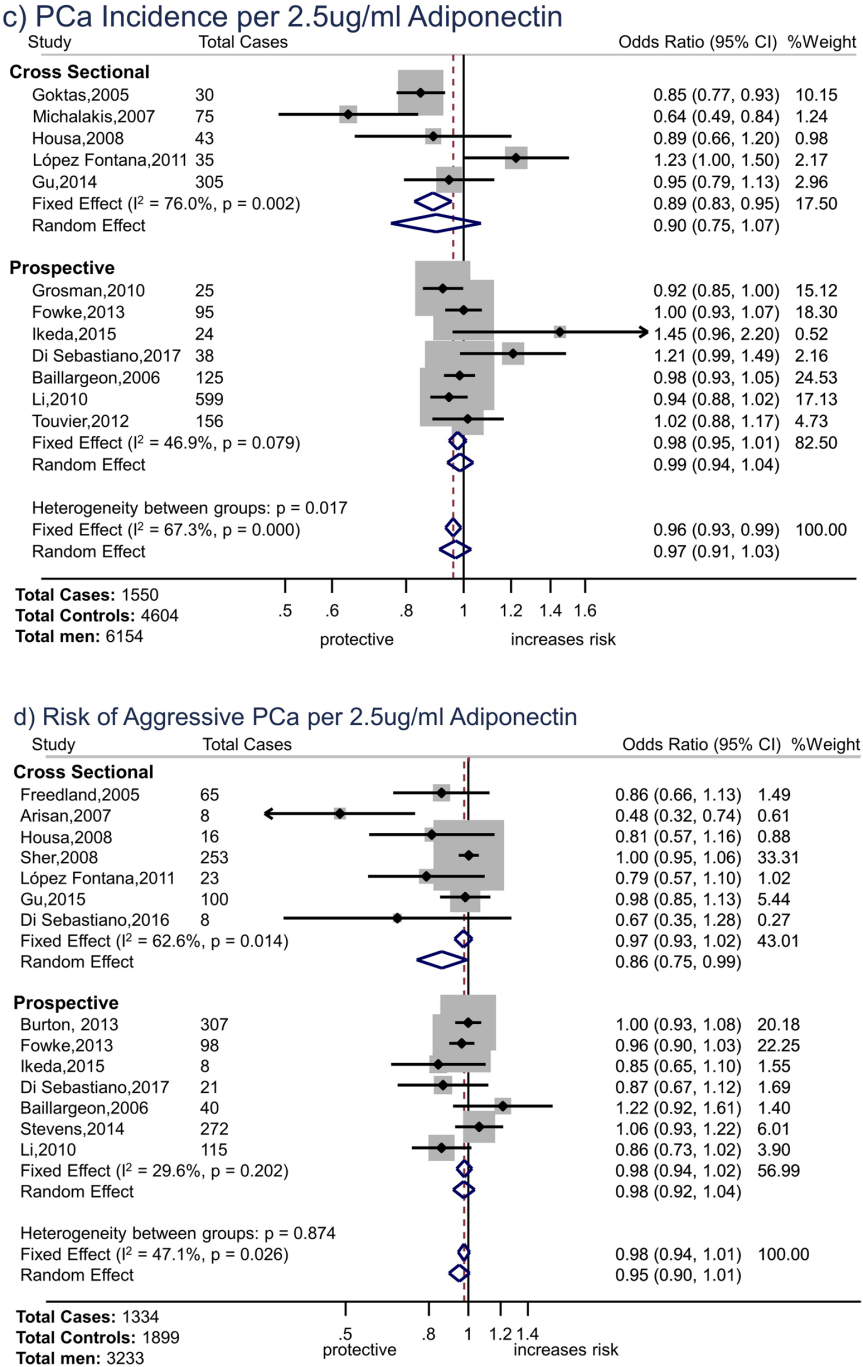
Forest Plots. (**a**) Forest plot of associations of leptin with total prostate cancer incidence (OR per 2.5 ng/ml increase in leptin) by study design. (**b**) Forest plot of associations of leptin with aggressive prostate cancer (OR per 2.5 ng/ml increase in leptin) by study design. (**c**) Forest plot of associations of adiponectin with total prostate cancer incidence (OR per 2.5 µg/ml increase in adiponectin) by study design. (**d**) Forest plot of associations of adiponectin with aggressive prostate cancer (OR per 2.5 µg/ml increase in adiponectin) by study design. *Notes* Ordered by date (cross-sectional studies) or mean time from blood draw to diagnosis (prospective studies). Weight for fixed-effects model.

For leptin and overall prostate cancer, the overall fixed effect OR was consistent with the null hypothesis (OR 1.00, 95% CI 0.98 to 1.02 per 2.5 ng/ml increase in leptin, p = 0.84) (Fig. 2a). However, there were considerable differences between study types: the pooled fixed effect OR for prospective studies was 0.97 (95% CI 0.95 to 0.99, p = 0.005) per 2.5 ng/ml increase in leptin, whereas that for cross-sectional studies was 1.19 (95% CI 1.13 to 1.26, p < 0.001). (Meta-regression p for difference in random effects estimates by study type p = 0.001). There was evidence of moderate heterogeneity amongst estimates from prospective studies (I^2^ = 54.9%) and cross-sectional studies (52.5%); therefore, pooling of individual study estimates may not be appropriate and these results should be interpreted cautiously.

There was weak evidence of a small association between leptin and aggressive prostate cancer (Fig. 2b, overall OR 1.03, 95% CI 1.00 to 1.06 per 2.5 ng/ml increase in leptin, p = 0.02). However, there was considerable heterogeneity amongst cross-sectional studies (I^2^ = 83.6%) for which the effect estimate was larger (pooled OR: 1.09 (95% CI 1.03 to 1.16), p = 0.004) than for prospective studies (OR: 1.02 (95% CI 0.99 to 1.05), p = 0.23, I^2^ 0.0%). (Meta-regression p for difference in random effects estimates by study type p = 0.27).

### Adiponectin

For adiponectin and overall prostate cancer, the overall pooled estimate indicated a small (4%) decreased risk of prostate cancer per 2.5 µg/ml increase in adiponectin (OR 0.96, 95% CI 0.93 to 0.99 per 2.5 µg/ml increase in adiponectin, p = 0.01) (Fig. 2c); the association was stronger in cross-sectional studies (OR 0.89, 95%CI 0.83 to 0.95, p = 0.001) than prospective studies (OR 0.98, 95% CI 0.95 to 1.01, p = 0.17, I^2^ = 46.9%), though there was considerable heterogeneity amongst the former (I^2^ = 76.0%). (Meta-regression p for difference in random effects estimates by study type p = 0.26).

Overall, there was little evidence of an association between adiponectin and aggressive prostate cancer (OR 0.98, 95%CI 0.94 to 1.01, p = 0.16, Fig. 2d). The pooled OR for cross-sectional studies was 0.97, 95% CI 0.93 to 1.02, p = 0.29, I^2^ = 62.6%. Most small (< 30 cases) cross-sectional studies found evidence that adiponectin was inversely associated with risk of aggressive prostate cancer, but larger studies did not. The prospective study estimates were more centred around the null with less evidence of heterogeneity (pooled OR 0.98 (95%CI 0.94 to 1.02, p = 0.33, I^2^ = 29.6%). (Meta-regression p for difference in random effects estimates by study type p = 0.34).

### Sensitivity analyses

The sensitivity analyses indicted that the pooled estimates were not sensitive to the majority of factors investigated (Supplementary Table S2). However, the weak association of leptin with aggressive prostate cancer was attenuated when maximally adjusted models were selected over minimally adjusted models. Redefining prospective as blood draw greater than one year before diagnosis did not change prospective study estimates. (Additionally, when stratified, there were no differences in pooled estimates from ‘diagnostic’ prospective studies compared to those where blood draw was greater than one year before diagnosis).

**Figure 3:**
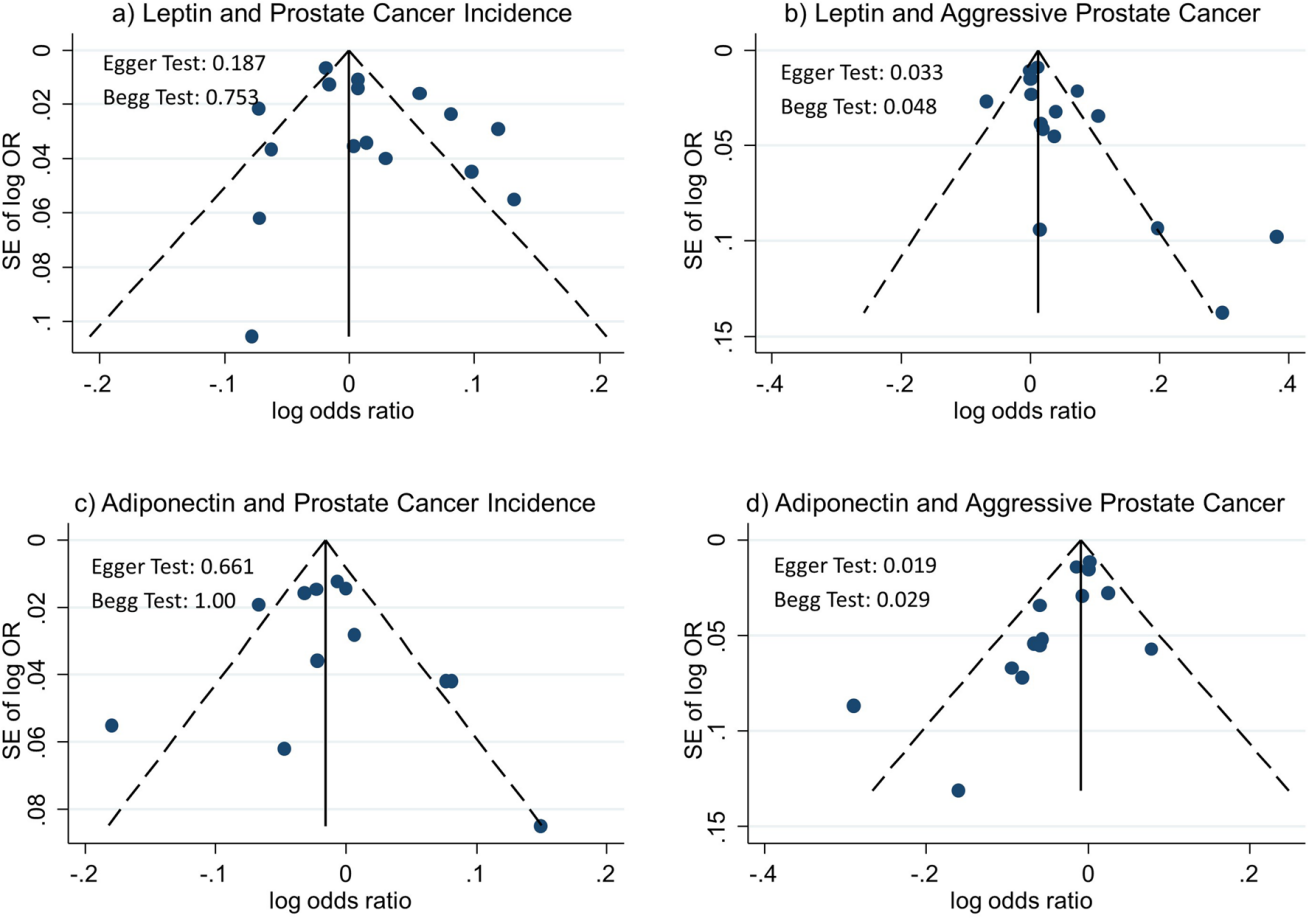
Funnel plots of included papers; adiponectin and leptin with incident or aggressive prostate cancer.

There was little evidence of funnel plot asymmetry in studies of associations of adiponectin or leptin with prostate cancer incidence (Fig. 3). However, there was some evidence of asymmetry in studies of aggressive prostate cancer; for leptin this asymmetry was to the right, or towards positive associations in smaller studies, and for adiponectin this was to the left, or towards inverse associations in smaller studies. These observations were supported by the Egger and Begg test results (p = 0.033 and p = 0.048 respectively for leptin and p = 0.019 and p = 0.029 respectively for adiponectin and aggressive prostate cancer).

### Influence analysis

In an influence analysis (Table 2), the overall results appeared relatively stable, particularly for prospective study pooled estimates. Exceptions are discussed. For leptin and prostate cancer incidence, exclusion of the study by Stocks et al.^58^) decreased the heterogeneity amongst prospective studies (from 54.9% to 27.4%) but slightly attenuated the affect estimate from 0.97 (0.95–0.99) to 0.98 (0.95–1.00). For leptin and aggressive prostate cancer, cross-sectional study estimates were affected by exclusion of Basaria et al.^40^: this attenuated the association from 1.09 (1.03–1.16) to 1.04 (0.97–1.12); and Freedland et al.^44^: this increased the estimate to 1.17 (1.09–1.25).

**Table 2:** Influence analysis.

Prostate cancer incidence							Aggressive prostate cancer						
	OR	LCI	UCI	Weight	P*	I^2^ (%)		OR	LCI	UCI	Weight	P*	I^2^ (%)
Cross-sectional							Cross-sectional						
**Leptin**													
All studies	1.19	1.13	1.26	14.3	< 0.001	52.5%	All studies	1.09	1.03	1.16	19.1	0.004	83.6%
Excluding							Excluding						
Lagiou, 1998	1.20	1.14	1.27	14.1	< 0.001	52.9%	Chang, 2001	1.07	1.00	1.14	17.1	0.06	84.3%
Hsing, 2001	1.19	1.12	1.26	13.7	< 0.001	56.0%	Saglam, 2003	1.08	1.01	1.14	18.8	0.02	80.1%
Saglam, 2003	1.19	1.11	1.26	11.4	< 0.001	59.6%	Basaria, 2005	1.04	0.97	1.12	13.7	0.25	84.4%
Basaria, 2005	1.19	1.12	1.26	13.5	< 0.001	59.2%	Freedland, 2005	1.17	1.09	1.25	15.7	< 0.001	75.4%
Gade-Andavolu, 2006	1.17	1.10	1.24	12.4	< 0.001	47.3%	Arisan, 2007	1.09	1.02	1.15	18.9	0.007	84.7%
Singh, 2010	1.24	1.15	1.34	7.9	< 0.001	54.5%	Singh, 2010	1.13	1.05	1.21	14.5	0.001	85.0%
López Fontana, 2011	1.21	1.14	1.28	13.8	< 0.001	29.7%	López Fontana, 2011	1.08	1.02	1.15	18.8	0.009	84.9%
							Di Sebastiano, 2016	1.09	1.03	1.16	18.8	0.004	86.0%
Prospective							Prospective						
All studies	0.97	0.95	0.99	85.8	0.005	54.9%	All studies	1.02	0.99	1.05	81.0	0.23	0.0%
Excluding							Excluding						
Baillargeon, 2006	0.97	0.95	0.99	84.0	0.01	60.3%	Baillargeon, 2006	1.02	0.99	1.05	80.6	0.25	0.0%
Stocks, 2007	0.98	0.95	1.00	85.2	0.03	27.4%	Stocks, 2007	1.02	0.99	1.05	80.6	0.26	0.0%
Li, 2010	0.96	0.94	0.99	84.3	0.002	54.5%	Li, 2010	1.02	0.99	1.05	80.4	0.33	0.0%
Neuhouser, 2010	0.98	0.95	1.01	76.0	0.26	56.2%	Neuhouser, 2010	1.01	0.97	1.05	70.6	0.56	0.0%
Touvier, 2012	0.97	0.95	0.99	85.6	0.004	60.1%	Burton, 2013	1.02	0.99	1.05	78.2	0.19	0.0%
Fowke, 2013	0.96	0.94	0.98	83.2	0.001	49.2%	Fowke, 2013	1.03	0.99	1.06	74.5	0.14	0.0%
Lai, 2014	0.97	0.95	0.99	85.6	0.009	56.0%	Di Sebastiano, 2017	1.02	0.99	1.05	80.7	0.27	0.0%
Gupta, 2016	0.97	0.95	0.99	85.5	0.004	59.1%							
Di Sebastiano, 2017	0.97	0.95	0.99	85.6	0.004	58.0%							
**Adiponectin**													
All studies	0.89	0.83	0.95	17.5	0.001	76.0%	All studies	0.97	0.93	1.02	43.0	0.29	62.6%
Excluding							Excluding						
Goktas, 2005	0.95	0.85	1.06	8.2	0.34	79.1%	Freedland, 2005	0.98	0.93	1.03	42.2	0.39	67.1%
Michalakis, 2007	0.91	0.84	0.98	16.5	0.01	71.6%	Arisan, 2007	0.98	0.93	1.03	42.7	0.51	10.2%
Housa, 2008	0.89	0.82	0.96	16.7	0.002	82.0%	Sher, 2008	0.88	0.79	0.97	14.6	0.02	55.4%
López Fontana, 2011	0.85	0.79	0.92	15.7	< 0.001	47.9%	Housa, 2008	0.98	0.93	1.03	42.5	0.37	66.8%
Gu, 2014	0.88	0.81	0.95	15.0	0.001	81.3%	López Fontana, 2011^27^	0.98	0.93	1.03	42.4	0.40	65.4%
							Gu, 2015	0.97	0.92	1.03	39.7	0.30	68.8%
							Di Sebastiano, 2016	0.98	0.93	1.03	42.9	0.34	66.1%
Prospective							Prospective						
All studies	0.98	0.95	1.01	82.5	0.17	46.9%	All studies	0.98	0.94	1.02	57.0	0.33	29.6%
Excluding							Excluding						
Baillargeon, 2006	0.97	0.94	1.01	76.8	0.20	55.5%	Baillargeon, 2006	0.97	0.93	1.02	56.4	0.23	18.8%
Grosman, 2010	0.99	0.95	1.03	79.4	0.58	43.0%							
Li, 2010	0.99	0.95	1.02	78.9	0.46	51.3%	Li, 2010	0.99	0.94	1.03	55.2	0.60	18.5%
Touvier, 2012	0.98	0.94	1.01	81.6	0.15	54.4%	Burton, 2013	0.97	0.92	1.02	46.1	0.23	37.6%
Fowke, 2013	0.97	0.94	1.01	78.6	0.13	53.7%	Fowke, 2013	0.99	0.93	1.05	44.7	0.66	39.5%
Ikeda, 2015	0.98	0.94	1.01	82.4	0.13	36.1%	Stevens, 2014	0.97	0.93	1.02	54.2	0.18	27.2%
Di Sebastiano, 2017	0.97	0.94	1.01	82.1	0.09	28.5%	Ikeda, 2015	0.98	0.94	1.03	56.3	0.44	31.8%
							Di Sebastiano, 2017	0.98	0.94	1.03	56.3	0.43	34.6%

Studies were sequentially excluded and analyses repeated. Pooled fixed-effects odds ratios. ORs per 2.5 ng/ml increase in leptin or 2.5 μg/ml increase in adiponectin.

*OR* odds ratio; *LCI* lower confidence interval; *UCI* upper confidence interval; *NA* not applicable.

For adiponectin and prostate cancer incidence, exclusion of one study by Lopez Fontana et al.^34^ reduced heterogeneity amongst cross-sectional studies from 76.0% to 47.9% but did not materially change the effect estimate. Exclusion of Goktas et al.^28^ attenuated the effect estimate from 0.89 (0.83–0.95) to 0.95 (0.85–1.06). In studies of aggressive prostate cancer, exclusion of Arisan et al.^39^ from cross-sectional study analyses did not affect the pooled estimate but reduced heterogeneity from 62.6% to 10.2%.

### Subgroup Analyses

Subgrouping analyses resulted in small numbers in groups so results are interpreted with caution (Supplementary Tables S3 and S4). Prospective studies were more likely to report odds ratios and cross-sectional studies mean differences. Stratification of studies by estimate type or assay type did not have a consistent effect on pooled estimates and estimates remained relatively consistent with the main findings. Where stage was reported as an outcome these results were pooled and compared to those for grade. For studies of leptin, a positive association with high grade, but not advanced stage, was seen. For studies of adiponectin, estimates were closer to the null for studies of stage than of grade. Prospective studies were more likely to report case detection by PSA screening; no cross-sectional studies reported case detection by PSA screening. Estimates were not different between those that were and were not screen-detected. However, where the PSA-screening status of patients was unclear, estimates were somewhat different to those from screen-detected or non-screen-detected men, possibly as this was a marker of the quality of study reporting.

Pooled effect estimates from studies of leptin or adiponectin did not appear to vary substantially when stratified by study-level mean BMI (Supplementary Fig. S1). There was little evidence of an interaction by mean study-level BMI (meta-regression p for difference in random effects estimates by mean BMI was > 0.3 in each main analysis).

Those studies that conducted BMI-stratified analyses (N = 7), did not provide evidence that the association of leptin with prostate cancer incidence or progression varied by BMI. None of the included studies examined adiponectin and prostate cancer incidence associations stratified by BMI. Two out of three studies that examined BMI-stratified associations of adiponectin with advanced stage or fatal prostate cancer found an inverse association in overweight men only (OR of advanced stage per log adiponectin unit in those with a BMI < 25: 1.48 (95%CI 0.77–2.82) and those with a BMI ≥ 25: 0.62 (0.42–0.90), p interaction: 0.006^17^; HR highest quintile of adiponectin compared to lowest quintile in those with BMI < 25: 0.86 (95%CI 0.31–2.38) and in those with BMI ≥ 25: 0.10 (95%CI 0.01–0.78), p for interaction 0.08^61^.

The search was updated to October 2018 and 103 new articles matching search criteria were identified. The abstract screen revealed 83 were ineligible (e.g. in vitro, genetic studies), and a further 15 were trials with no control groups, repeat publications or abstracts. 5 studies were eligible^62–66^. The albatross plots^26^ in Supplementary Fig. S2 indicate no strong consistent effects in either direction, with larger studies clustered around the null (no association) and smaller studies reporting smaller p values (stronger associations). For leptin, these small study effects were in the direction of a positive association and for adiponectin a negative association. There is no obvious bias from exclusion of these studies.

## Discussion

No strong, consistent associations between adipokines levels and risk of incident or aggressive prostate cancer were found. Pooled effect estimates from cross-sectional studies tended to be larger, more heterogenous and less stable to sensitivity analyses than those from prospective studies. When just the prospective evidence was considered, all pooled effect estimates were consistent with the null hypothesis accept for a weak inverse association between leptin and overall prostate cancer (3% decreased risk per 2.5 ng/ml increase in leptin).

A mainly qualitative systematic review of adiponectin, leptin and ghrelin levels with prostate cancer incidence and advanced disease was recently published^67^. It included a small exploratory meta-analysis and, in general, their findings corresponded with ours. However, they reported some suggestive evidence of an inverse association between adiponectin and advanced prostate cancer (meta relative risk 0.81 (95%CI 0.61–1.08) comparing the highest subset of adiponectin). This was based on 4 nested case–control studies, one which was included twice. Our pooled effect estimate for adiponectin and aggressive prostate cancer, which was derived from 7 prospective and 7 cross-sectional studies, did not indicate evidence of an inverse dose–response association (OR 0.98, 95%CI 0.94–1.01). A meta-analysis of genetic polymorphisms in adiponectin, leptin and their receptors found several associations with prostate cancer risk and aggressiveness^68^. Although this provides evidence that adipokine signaling may be involved in prostate carcinogenesis, the analyses were limited by the number of studies included. Additionally, studies of circulating adipokines in the general populations cannot exclude the possibility that adipokines may be associated with prostate cancer in certain subgroups of the population, such as those with particular metabolic profiles, or through local paracrine signalling of adipokines due to the abundance of periprostatic adipose tissue.

Relevant case-control studies nested within the San Antonio Center for Biomarkers of Risk of Prostate Cancer cohort study were reported in two papers. Baillargeon et al.^38^ included fewer cases and controls but examined both leptin and adiponectin, measured by multi-analyte profiling. Medina et al.^69^ focused on adiponectin multimers, measured by ELISA. They found only high-molecular weight adiponectin was associated with prostate cancer incidence (but not total, middle- or low-molecular weight adiponectin). The decision was taken to include the former as it was deemed more comparable with the other studies, but inclusion of the later would not have altered the results.

### Limitations

Many studies were small and quality of reporting was variable. There was considerable heterogeneity amongst study estimates, particularly cross-sectional evidence, indicating the true effect estimates may vary between studies. Confounding and selection bias are inherent issues in observational data; as a result meta-analysis of such data risks producing spuriously precise pooled effect estimates (Egger et al. 2001) Consequently, it is recommended that the quantitative results should be interpreted with caution, and sources of heterogeneity thoroughly explored^70^. However, the number of studies was too small, and the detail given in some reports insufficient, to explore heterogeneity adequately. There was heterogeneity in the definition of case and control groups; in particular, for aggressive prostate cancer. The majority used Gleason score as an outcome, although some included Gleason 7 as high grade and others did not—a matter that has been much debated in the literature. As a result of the mixture of outcome definitions, some studies may have included men with Gleason 7 in the comparison (‘non-aggressive’) group, potentially leading to an attenuation of any association. There was some evidence of possible reporting bias in studies of adipokines and aggressive prostate cancer, although there are other potential causes of funnel plot asymmetry such as poor study methodology^71^. Non-linear trends could not be assessed due to the limited number and quality of studies. The detection of prostate cancer is complex and it is recognised that PSA-screening can result in over-diagnosis; we therefore performed a subgroup analysis in which studies based on PSA-screening were meta-analysed separately from those in which cancers were clinically detected. However, several studies did not provide information on how cases were identified, preventing proper exploration of the effect of PSA-screening on associations.

Obesity or hormones such as insulin-like growth factor (IGF)-I may be on the causal pathway and therefore adjustment for such factors (mediators) could lead to over-adjustment. Adiposity may interact with the association of adiponectin and leptin with prostate cancer^49^; therefore, associations should ideally be examined separately in normal weight and overweight men. This may be particularly important for associations of adiponectin with prostate cancer as adiponectin is an insulin-sensitising hormone and may have a more marked effect in men at higher risk of insulin resistance^17^. Two out of three studies that did include analyses of adiponectin with aggressive prostate cancer stratified by BMI, found an inverse association with higher prostate cancer stage^17^, and fatal prostate cancer^61^, respectively, in obese and overweight men only. Medina et al.^69^ reported that the association of high-molecular weight adiponectin with prostate cancer incidence was adiposity dependent; positive in normal and overweight men and inverse in obese men. We further explored this by conducting subgroup analyses stratifying by the mean study-level BMI. We did not find further evidence to support this interaction with adiposity. Although an advantage of observational studies is that population subgroups often excluded from trials are more likely to be included^70^, very few black men were studied. This is particularly important in prostate cancer, as black men are around 2–3 times more likely to develop the disease than white men^72^. Therefore, this review cannot be considered representative of the wider population at risk.

Dose–response meta-analysis assumes a linear relationship between the exposure and outcome. The association of adiponectin and leptin with prostate cancer stage may be U-shaped^17^ (possibly due to opposite patterns of association in overweight and normal weight men) and therefore such analysis may fail to detect an important association. The possibility of reverse causality in these studies (the disease state affecting adipokine levels) cannot be excluded. Finally, and perhaps most importantly, observational data cannot identify causal associations because of the considerable possibility of confounding; any associations found may be markers of another, unmeasured, factor. For example, insulin resistance is a risk factor for prostate cancer and, as adiponectin is an insulin-sensitising hormone, levels are lower in men with insulin resistance.

### Strengths

This review was not limited to English-only papers (although no non-English language papers were identified), or papers that reported one type of estimate. Several authors were contacted to request further information so their data could be included and the response rate to these requests was high. This is important as the strength of an association can affect the amount of detail reported and therefore the likelihood of being able to extract enough data to derive a dose–response OR, which can introduce bias. Our comprehensive search strategy and flexibility in deriving a dose–response OR from limited data will also have helped reduce this bias. Only 3 studies were excluded as the estimates could not be extracted or converted to a plausible dose–response OR. Stratification by point of data collection/study type (cross-sectional versus prospective) provided a means to explore the possibility of reverse causality. It appeared that for leptin in particular, reverse causality or selection bias is a possibility and prospective data may provide a more reliable estimate. Leptin and adiponectin are stable over at least 6 freeze thaw cycles^73,74^. They exhibit slight diurnal variation but levels are relatively stable throughout the day (reaching a nadir at night)^75^ and over time (the intra-class correlation coefficient derived from 4 samples taken over 1 year was 0.74 for leptin and 0.81 for adiponectin^76^. However, the variation associated with a single measure will tend to bias results towards the null; therefore, associations will not be overestimated.

## Conclusion

We did not find strong evidence to support our hypotheses that leptin is positively associated with risk of overall and aggressive prostate cancer and adiponectin is inversely associated with risk of overall and aggressive prostate cancer. The weak evidence that leptin is inversely associated with overall prostate cancer risk reflects the findings of the Mendelian randomisation analysis of weak evidence of an association of BMI with lower prostate cancer risk^10^, but it is not possible to know whether the association of BMI is mediated by leptin or leptin is purely a marker for high BMI. On an individual level, as a biomarker for detection or prognosis, adiponectin or leptin are unlikely to be useful as potential screening tools require exceptionally high ORs to give acceptable detection rates^77^.

## Supplementary Information

Supplementary Information.

## Data Availability

The study is a systematic review and meta-analysis. All of the data are available from the studies listed in in the Table 1.

## Abbreviations

BMI: Body Mass Index
IGF: Insulin-like Growth factor
OR: Odds ratio
CI: Confidence interval
PSA: Prostate-specific antigen
ELISA: Enzyme immunoassay
RIA: Radioimmunoassay
MAP: Multi-analyte processing
LTIA: Latex particle-enhanced turbidimetric immunoassay
