# Supplementary material for: Circulating adiponectin and leptin and risk of overall and aggressive prostate cancer: a systematic review and meta-analysis
Source: Sci Rep. 2021 Jan 11;11:320. doi: 10.1038/s41598-020-79345-4 (PMC7801499; doi:10.1038/s41598-020-79345-4)

**Supplementary Table S1: Search Terms**

| **Ovid Search Terms (used to search Medline and Embase)** | **Web of Knowledge Search Terms (used to search Web of Science and BIOSIS Previews)** |
| --- | --- |
| 1. Prostatic Neoplasms/ | 1 TS=(prosta* SAME neoplas*) |
| 2. (prostat* adj3 cancer$).tw | 2 TS=(prostat* SAME tumo$r*) |
| 3. (prostat* adj3 carcinoma$).tw. | 3 TS=(prostat*SAME carcinoma*) |
| 4. (prostat* adj3 tumo?r$).tw. | 4 TS=(prostat* SAME cancer*) |
| 5. (prosta* adj3 neoplas$).tw. | 5 TS=(ob protein) |
| 6. adiponectin/ | 6 TS=(obese protein) |
| 7. adiponectin.tw. | 7 TS=(leptin) |
| 8. GBP.tw. | 8 TS=(acrp30) |
| 9. GBP?28.tw. | 9 TS=(apm1) |
| 10. gelatin binding protein.tw. | 10 TS=(gelatin binding protein) |
| 11. apm1.tw. | 11 TS=(adiponectin) |
| 12. acrp30.tw. | 12 #4 OR #3 OR #2 OR #1 |
| 13. leptin/ | 13 #11 OR #10 OR #9 OR #8 OR #7 OR #6 OR #5 |
| 14. obese protein.tw. | 14 #13 AND #12 |
| 15. leptin.tw. |  |
| 16. ob protein.tw. |  |
| 17. 1 or 2 or 3 or 4 or 5 |  |
| 18. 6 or 7 or 8 or 9 or 10 or 11 or 12 |  |
| 19. 13 or 14 or 15 or 16 |  |
| 20. 18 or 19 |  |
| 21. 17 and 20 |  |

**Supplementary Table S2 Sensitivity Analyses**

|  |  | **Main Analysis** | | | | **Sensitivity Analysis 1** | | | | **Sensitivity Analysis 2** | | | | **Sensitivity Analysis 3** | | | |  | **Sensitivity Analysis 4** | | | |
| --- | --- | --- | --- | --- | --- | --- | --- | --- | --- | --- | --- | --- | --- | --- | --- | --- | --- | --- | --- | --- | --- | --- |
|  | No. of Studies | **OR** | **LCI** | **UCI** | **I^2^** | **OR** | **LCI** | **UCI** | **I^2^** | **OR** | **LCI** | **UCI** | **I^2^** | **OR** | **LCI** | **UCI** | **I^2^** | No. of Studies | **OR** | **LCI** | **UCI** | **I^2^** |
| **LEPTIN** |  |  |  |  |  |  |  |  |  |  |  |  |  |  |  |  |  |  |  |  |  |  |
| INCIDENCE |  |  |  |  |  |  |  |  |  |  |  |  |  |  |  |  |  |  |  |  |  |  |
| Prospective | 9 | **0.97** | 0.95 | 0.99 | 54.9% | **0.98** | 0.95 | 1.00 | 54.6% |  |  |  |  |  |  |  |  | 7 | **0.96** | 0.93 | 0.98 | 51.9% |
| Cross-Sectional | 7 | **1.19** | 1.13 | 1.26 | 52.5% | **1.18** | 1.12 | 1.25 | 49.1% |  |  |  |  |  |  |  |  | 9 | **1.10** | 1.06 | 1.14 | 73.2% |
| Overall | 16 | **1.00** | 0.98 | 1.02 | 80.9% | **1.01** | 0.99 | 1.04 | 77.7% |  |  |  |  |  |  |  |  | 16 | **1.00** | 0.98 | 1.02 | 80.9% |
| AGGRESSIVE |  |  |  |  |  |  |  |  |  |  |  |  |  |  |  |  |  |  |  |  |  |  |
| Prospective | 7 | **1.02** | 0.99 | 1.05 | 0.0% | **1.00** | 0.97 | 1.04 | 0.0% | **1.02** | 0.99 | 1.05 | 0.0% | **1.00** | 0.97 | 1.03 | 0.0% | 4 | **1.03** | 0.99 | 1.08 | 0.00% |
| Cross-Sectional | 8 | **1.09** | 1.03 | 1.16 | 83.6% | **1.09** | 1.03 | 1.16 | 83.6% | **1.10** | 1.04 | 1.17 | 82.8% | **1.10** | 1.04 | 1.17 | 82.8% | 11 | **1.03** | 1.00 | 1.07 | 79.5% |
| Overall | 15 | **1.03** | 1.01 | 1.06 | 71.7% | **1.02** | 0.99 | 1.05 | 71.9% | **1.03** | 1.01 | 1.06 | 71.2% | **1.02** | 1.00 | 1.05 | 72.8% | 15 | **1.03** | 1.01 | 1.06 | 71.7% |
| **ADIPONECTIN** |  |  |  |  |  |  |  |  |  |  |  |  |  |  |  |  |  |  |  |  |  |  |
| INCIDENCE |  |  |  |  |  |  |  |  |  |  |  |  |  |  |  |  |  |  |  |  |  |  |
| Prospective | 7 | **0.98** | 0.95 | 1.01 | 76.0% | **0.98** | 0.95 | 1.02 | 44.1% |  |  |  |  |  |  |  |  | 3 | **0.97** | 0.93 | 1.02 | 0.0% |
| Cross-Sectional | 5 | **0.89** | 0.83 | 0.95 | 46.9% | **0.89** | 0.83 | 0.96 | 74.3% |  |  |  |  |  |  |  |  | 9 | **0.95** | 0.91 | 0.99 | 74.9% |
| Overall | 12 | **0.96** | 0.93 | 0.99 | 67.3% | **0.96** | 0.93 | 1.00 | 65.0% |  |  |  |  |  |  |  |  | 12 | **0.96** | 0.93 | 0.99 | 67.3% |
| AGGRESSIVE |  |  |  |  |  |  |  |  |  |  |  |  |  |  |  |  |  |  |  |  |  |  |
| Prospective | 7 | **0.98** | 0.94 | 1.02 | 29.6% | **0.97** | 0.93 | 1.01 | 67.6% | **0.98** | 0.93 | 1.02 | 49.4% | **0.98** | 0.94 | 1.03 | 0.0% | 3 | **1.01** | 0.91 | 1.11 | 65.0% |
| Cross-Sectional | 7 | **0.97** | 0.93 | 1.02 | 62.6% | **0.98** | 0.92 | 1.03 | 63.7% | **1.00** | 0.95 | 1.05 | 69.7% | **1.00** | 0.95 | 1.06 | 70.0% | 11 | **0.97** | 0.94 | 1.01 | 45.9% |
| Overall | 14 | **0.98** | 0.94 | 1.01 | 47.1% | **0.97** | 0.94 | 1.01 | 63.0% | **0.98** | 0.95 | 1.02 | 59.3% | **0.99** | 0.96 | 1.03 | 49.4% | 14 | **0.98** | 0.94 | 1.01 | 47.1% |

**Notes:** Fixed-effects estimates. ORs per 2.5 ng/ml increase in leptin or 2.5 μg/ml increase in adiponectin

Abbreviations: OR, Odds Ratio; LCI, Lower 95% Confidence Interval; UCI, Upper 95% Confidence Interval, I2, I squared statistic (an index of heterogeneity)

Main Analysis: Grade>Stage>Other measures of aggressiveness and Minimally adjusted estimates > maximally adjusted.

Sensitivity Analysis 1: Maximally adjusted estimates>minimally adjusted and Grade>Stage>Other measures of aggressiveness

Sensitivity Analysis 2: Stage>Other measures of aggressiveness>Grade and Minimally adjusted estimates>maximally adjusted

Sensitivity Analysis 3: Stage>Other measures of aggressiveness>Grade and Maximally adjusted estimates > minimally adjusted

Sensitivity Analysis 4: As main analysis, but prospective redefined as blood draw ≥1 year before to diagnosis.

**Supplementary Table S3 Subgroup Analyses: Leptin**

|  | **INCIDENCE** | | | | | | **AGGRESSIVE** | | | | | |
| --- | --- | --- | --- | --- | --- | --- | --- | --- | --- | --- | --- | --- |
|  | No. Studies | **OR** | LCI | UCI | % Weight | I^2^ (%) | No. Studies | **OR** | LCI | UCI | % Weight | I^2^ (%) |
| **Mean Difference** |  |  |  |  |  |  |  |  |  |  |  |  |
| Prospective | 3 | **1.02** | 0.97 | 1.07 | 61.3 | 0.0% | 2 | **1.00** | 0.95 | 1.06 | 58.3 | 0.0% |
| Cross-Sectional | 4 | **1.17** | 1.10 | 1.24 | 38.7 | 51.0% | 8 | **1.09** | 1.03 | 1.16 | 41.7 | 83.6% |
| **OR** |  |  |  |  |  |  |  |  |  |  |  |  |
| Prospective | 6 | **0.96** | 0.93 | 0.98 | 96.0 | 56.8% | 5 | **1.03** | 0.99 | 1.06 | 100.0 | 0.0% |
| Cross-Sectional | 3 | **1.32** | 1.17 | 1.49 | 4.0 | 41.7% | 0 | **NA** |  |  |  |  |
|  |  |  |  |  |  |  |  |  |  |  |  |  |
| **RIA** |  |  |  |  |  |  |  |  |  |  |  |  |
| Prospective | 4 | **0.99** | 0.96 | 1.03 | 83.3 | 75.1% | 2 | **1.01** | 0.96 | 1.06 | 91.7 | 0.0% |
| Cross-Sectional | 4 | **1.27** | 1.17 | 1.38 | 16.7 | 27.4% | 3 | **1.40** | 1.20 | 1.65 | 8.3 | 85.9% |
| **ELISA** |  |  |  |  |  |  |  |  |  |  |  |  |
| Prospective | 4 | **0.96** | 0.93 | 0.99 | 84.4 | 5.5% | 3 | **1.02** | 0.98 | 1.06 | 75.1 | 0.0% |
| Cross-Sectional | 3 | **1.14** | 1.06 | 1.23 | 15.6 | 60.8% | 6 | **1.05** | 0.98 | 1.12 | 24.9 | 79.8% |
| **MAP or LTIA** |  |  |  |  |  |  |  |  |  |  |  |  |
| Prospective | 1 | 0.96 | 0.90 | 1.02 | 100.0 | NA | 1 | **1.04** | 0.86 | 1.26 | 100.0 | NA |
| Cross-Sectional | 0 | **NA** |  |  |  |  | 0 | **NA** |  |  |  |  |
|  |  |  |  |  |  |  |  |  |  |  |  |  |
| **Stage** |  |  |  |  |  |  |  |  |  |  |  |  |
| Prospective |  |  |  |  |  |  | 1 | **1.00** | 0.93 | 1.08 | 69.4 | NA |
| Cross-Sectional |  |  |  |  |  |  | 3 | **1.01** | 0.90 | 1.12 | 30.6 | 92.5% |
| **Grade** |  |  |  |  |  |  |  |  |  |  |  |  |
| Prospective |  |  |  |  |  |  | 6 | **1.02** | 0.99 | 1.05 | 92.8 | 0.0% |
| Cross-Sectional |  |  |  |  |  |  | 4 | **1.10** | 0.99 | 1.22 | 7.2 | 85.8% |
| **Combination/other** |  |  |  |  |  |  |  |  |  |  |  |  |
| Prospective |  |  |  |  |  |  | 2 | **1.07** | 0.94 | 1.22 | 31.2 | 0.0% |
| Cross-Sectional |  |  |  |  |  |  | 3 | **1.22** | 1.12 | 1.33 | 68.78 | 0.0% |
|  |  |  |  |  |  |  |  |  |  |  |  |  |
| **PSA screen-detected†** |  |  |  |  |  |  |  |  |  |  |  |  |
| Prospective | 2 | **0.96** | 0.93 | 0.98 | 100.0 | 0.0% | 3 | **1.02** | 0.98 | 1.06 | 100.0 | 0.0% |
| Cross-Sectional | 0 | NA |  |  |  |  | 0 | **NA** |  |  |  |  |
| **Not PSA screen-detected** |  |  |  |  |  |  |  |  |  |  |  |  |
| Prospective | 5 | **0.99** | 0.95 | 1.02 | 69.5 | 29.7% | 3 | **1.01** | 0.96 | 1.06 | 70.6 | 0.0% |
| Cross-Sectional | 6 | **1.21** | 1.14 | 1.28 | 30.5 | 72.5% | 5 | **1.14** | 1.05 | 1.22 | 29.4 | 80.2% |
| **Not reported** |  |  |  |  |  |  |  |  |  |  |  |  |
| Prospective | 2 | **1.04** | 0.91 | 1.18 | 84.6 | 0.0% | 1 | **1.10** | 0.88 | 1.37 | 17.3 | NA |
| Cross-Sectional | 1 | **0.84** | 0.62 | 1.13 | 15.4 | NA | 3 | **1.02** | 0.92 | 1.13 | 82.7 | 89.9% |

Notes: Fixed effects estimates. ORs per 2.5 ng/ml increase in leptin † Cases identified through PSA-screening. Abbreviations: NA, Not Applicable; OR, Odds Ratio; LCI, Lower 95% Confidence Interval; UCI, Upper 95% Confidence Interval, I2, I squared statistic (an index of heterogeneity)

**Supplementary Table S4 Subgroup Analyses: Adiponectin**

|  | **INCIDENCE** | | | | | | **AGGRESSIVE** | | | | | |  |
| --- | --- | --- | --- | --- | --- | --- | --- | --- | --- | --- | --- | --- | --- |
|  | No. Studies | **OR** | LCI | UCI | % Weight | I^2^ (%) | No. Studies | **OR** | LCI | UCI | % Weight | I^2^ (%) | |
| **Mean Difference** |  |  |  |  |  |  |  |  |  |  |  |  | |
| Prospective | 4 | **0.98** | 0.94 | 1.03 | 69.0 | 70.2% | 3 | **0.95** | 0.89 | 1.02 | 75.6 | 0.0% | |
| Cross-Sectional | 4 | **0.91** | 0.84 | 0.98 | 31.1 | 71.6% | 5 | **0.88** | 0.78 | 0.99 | 24.4 | 64.2% | |
| **OR** |  |  |  |  |  |  |  |  |  |  |  |  | |
| Prospective | 3 | **0.97** | 0.93 | 1.02 | 97.4 | 0.0% | 4 | **1.00** | 0.95 | 1.06 | 47.5 | 47.6% | |
| Cross-Sectional | 1 | **0.64** | 0.49 | 0.84 | 2.6 | NA | 2 | **1.00** | 0.94 | 1.06 | 52.5 | 15.5% | |
|  |  |  |  |  |  |  |  |  |  |  |  |  | |
| **RIA** |  |  |  |  |  |  |  |  |  |  |  |  | |
| Prospective | 3 | **0.96** | 0.92 | 1.00 | 80.3 | 16.6% | 2 | **0.95** | 0.89 | 1.01 | 96.7 | 33.0% | |
| Cross-Sectional | 3 | **0.83** | 0.76 | 0.90 | 19.7 | 49.1% | 1 | **0.81** | 0.57 | 1.16 | 3.3 | NA | |
| **ELISA** |  |  |  |  |  |  |  |  |  |  |  |  | |
| Prospective | 2 | **1.07** | 0.96 | 1.21 | 57.3 | 47.8% | 3 | **1.01** | 0.94 | 1.07 | 39.8 | 0.0% | |
| Cross-Sectional | 2 | **1.06** | 0.92 | 1.21 | 42.7 | 71.9% | 6 | **0.98** | 0.93 | 1.03 | 60.2 | 66.8% | |
| **MAP or LTIA** |  |  |  |  |  |  |  |  |  |  |  |  | |
| Prospective | 2 | **0.99** | 0.93 | 1.05 | 100.0 | 69.5% | 2 | **1.00** | 0.83 | 1.22 | 100.0 | 70.6% | |
| Cross-Sectional | 0 | **NA** |  |  |  |  | 0 | **NA** |  |  |  |  | |
|  |  |  |  |  |  |  |  |  |  |  |  |  | |
| **Stage** |  |  |  |  |  |  |  |  |  |  |  |  | |
| Prospective |  |  |  |  |  |  | 1 | **1.00** | 0.93 | 1.08 | 77.6 | NA | |
| Cross-Sectional |  |  |  |  |  |  | 4 | **1.02** | 0.89 | 1.17 | 22.4 | 81.6% | |
| **Grade** |  |  |  |  |  |  |  |  |  |  |  |  | |
| Prospective |  |  |  |  |  |  | 5 | **0.97** | 0.93 | 1.02 | 57.0 | 31.3% | |
| Cross-Sectional |  |  |  |  |  |  | 5 | **0.97** | 0.92 | 1.03 | 43.0 | 72.8% | |
| **Combination/other** |  |  |  |  |  |  |  |  |  |  |  |  | |
| Prospective |  |  |  |  |  |  | 2 | **0.95** | 0.85 | 1.05 | 63.8 | 74.3% | |
| Cross-Sectional |  |  |  |  |  |  | 3 | **0.97** | 0.84 | 1.11 | 36.2 | 22.7% | |
|  |  |  |  |  |  |  |  |  |  |  |  |  | |
| **PSA screen-detected** † |  |  |  |  |  |  |  |  |  |  |  |  | |
| Prospective | 3 | **0.97** | 0.92 | 1.01 | 100.0 | 62.5% | 3 | **1.00** | 0.93 | 1.07 | 100.0 | 41.2% | |
| Cross-Sectional | 0 | **NA** |  |  |  |  | 0 | **NA** |  |  |  |  | |
| **Not PSA screen-detected** |  |  |  |  |  |  |  |  |  |  |  |  | |
| Prospective | 2 | **0.97** | 0.93 | 1.02 | 69.8 | 15.2% | 2 | **0.95** | 0.89 | 1.01 | 78.4 | 33.0% | |
| Cross-Sectional | 4 | **0.85** | 0.79 | 0.92 | 30.2 | 47.9% | 4 | **0.89** | 0.79 | 1.01 | 21.6 | 72.0% | |
| **Not reported** |  |  |  |  |  |  |  |  |  |  |  |  | |
| Prospective | 2 | **1.07** | 0.96 | 1.21 | 76.1 | 47.8% | 2 | **1.02** | 0.90 | 1.15 | 17.7 | 47.9% | |
| Cross-Sectional | 1 | **1.23** | 1.00 | 1.51 | 24.0 | NA | 3 | **0.99** | 0.94 | 1.05 | 82.3 | 34.5% | |

Notes: Fixed effects estimates. ORs per 2.5 ng/ml increase in leptin † Cases identified through PSA-screening. Abbreviations: NA, Not Applicable; OR, Odds Ratio; LCI, Lower 95% Confidence Interval; UCI, Upper 95% Confidence Interval, I2, I squared statistic (an index of heterogeneity)

**Supplementary Figure S1 Subgroup Analysis by mean study-level BMI**


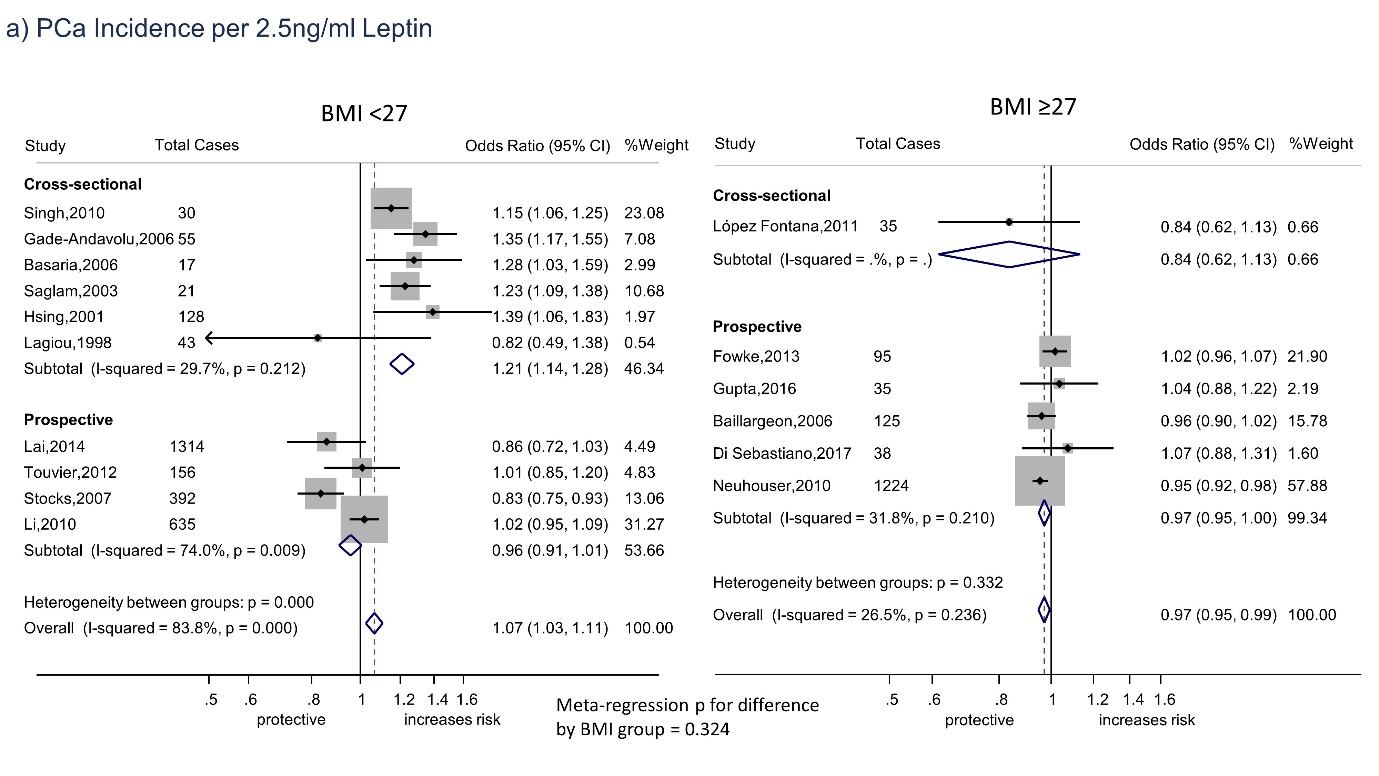


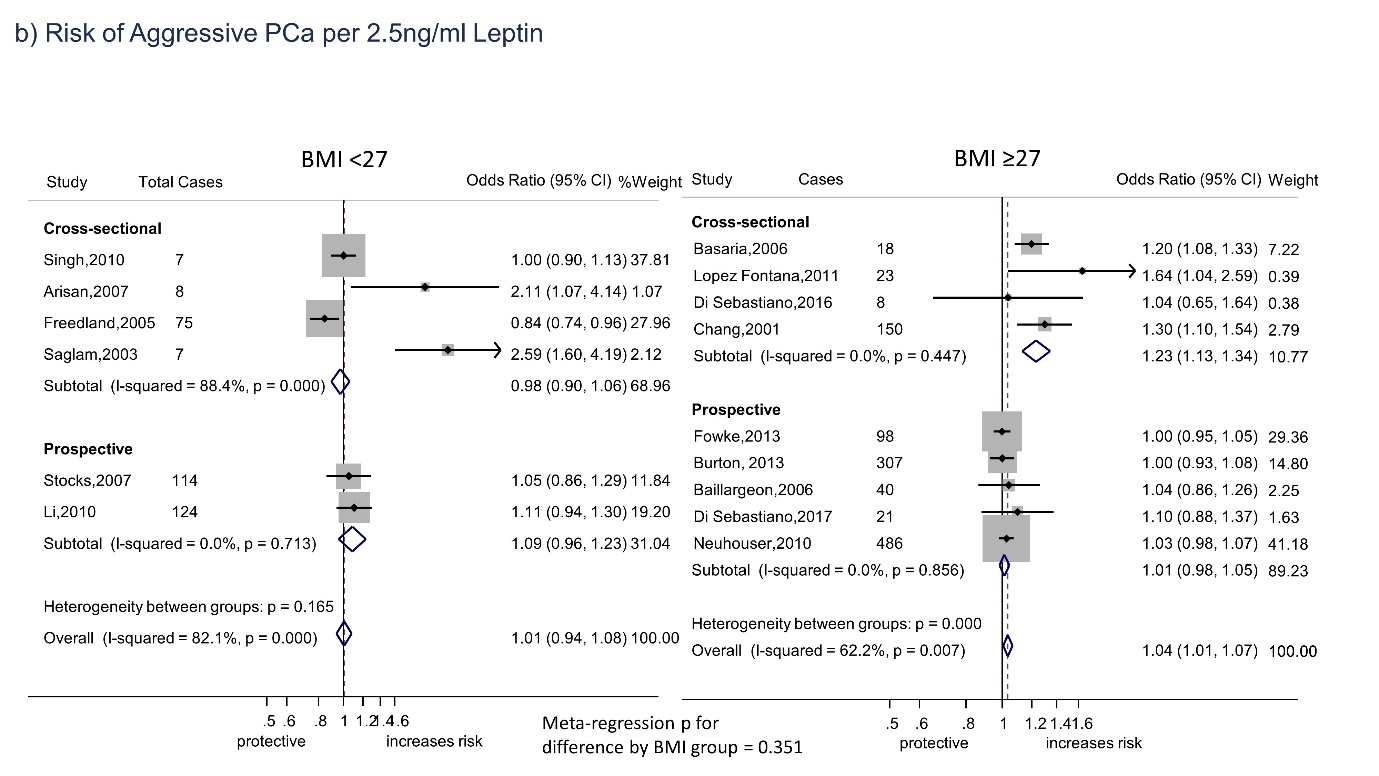


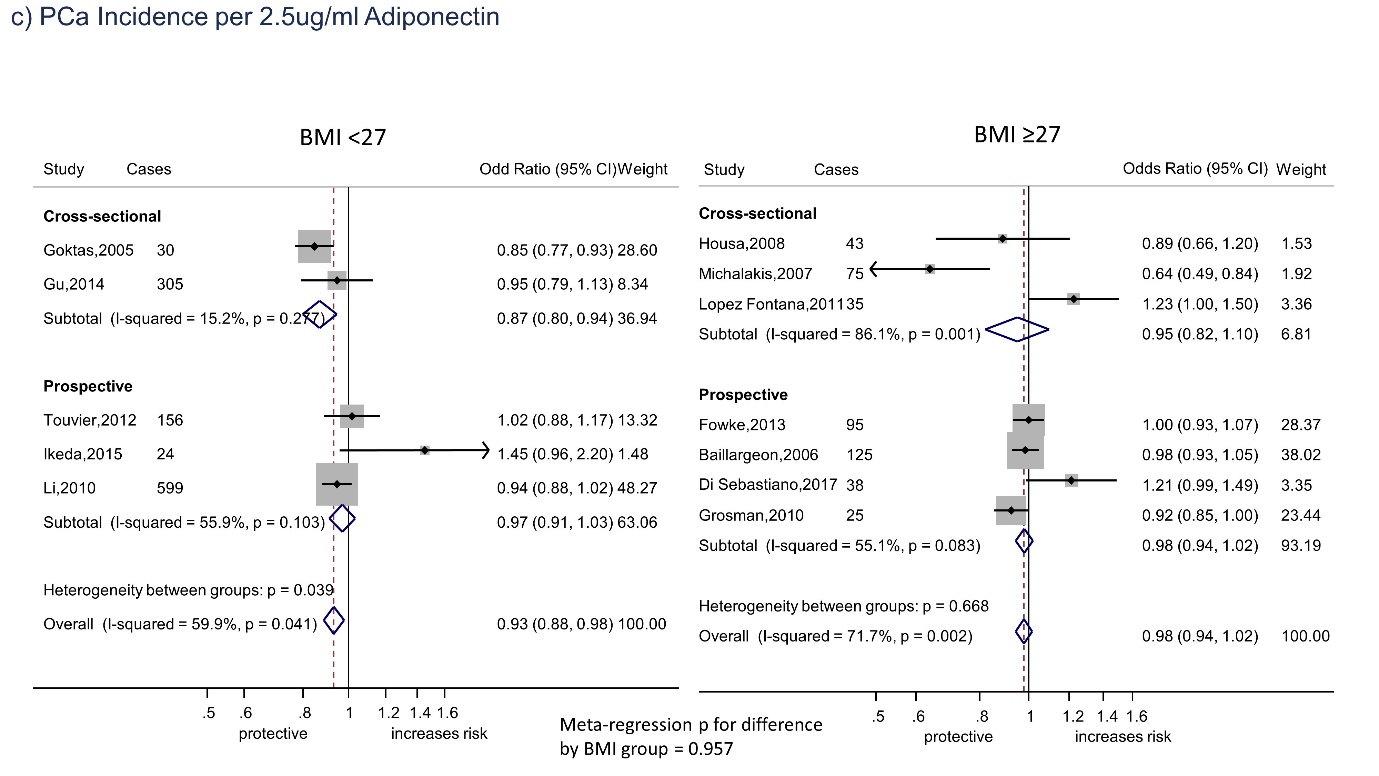

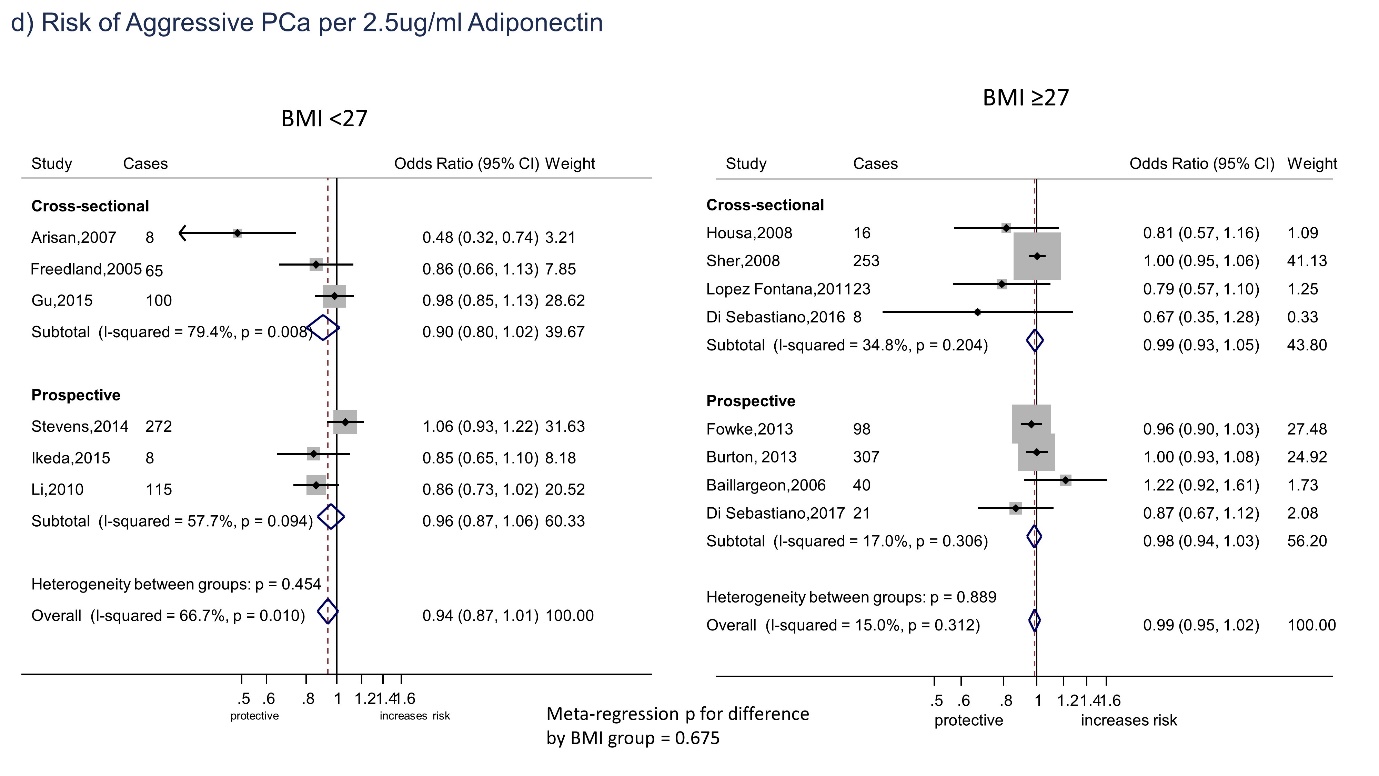


**Supplementary Figure S2 Albatross Plots**


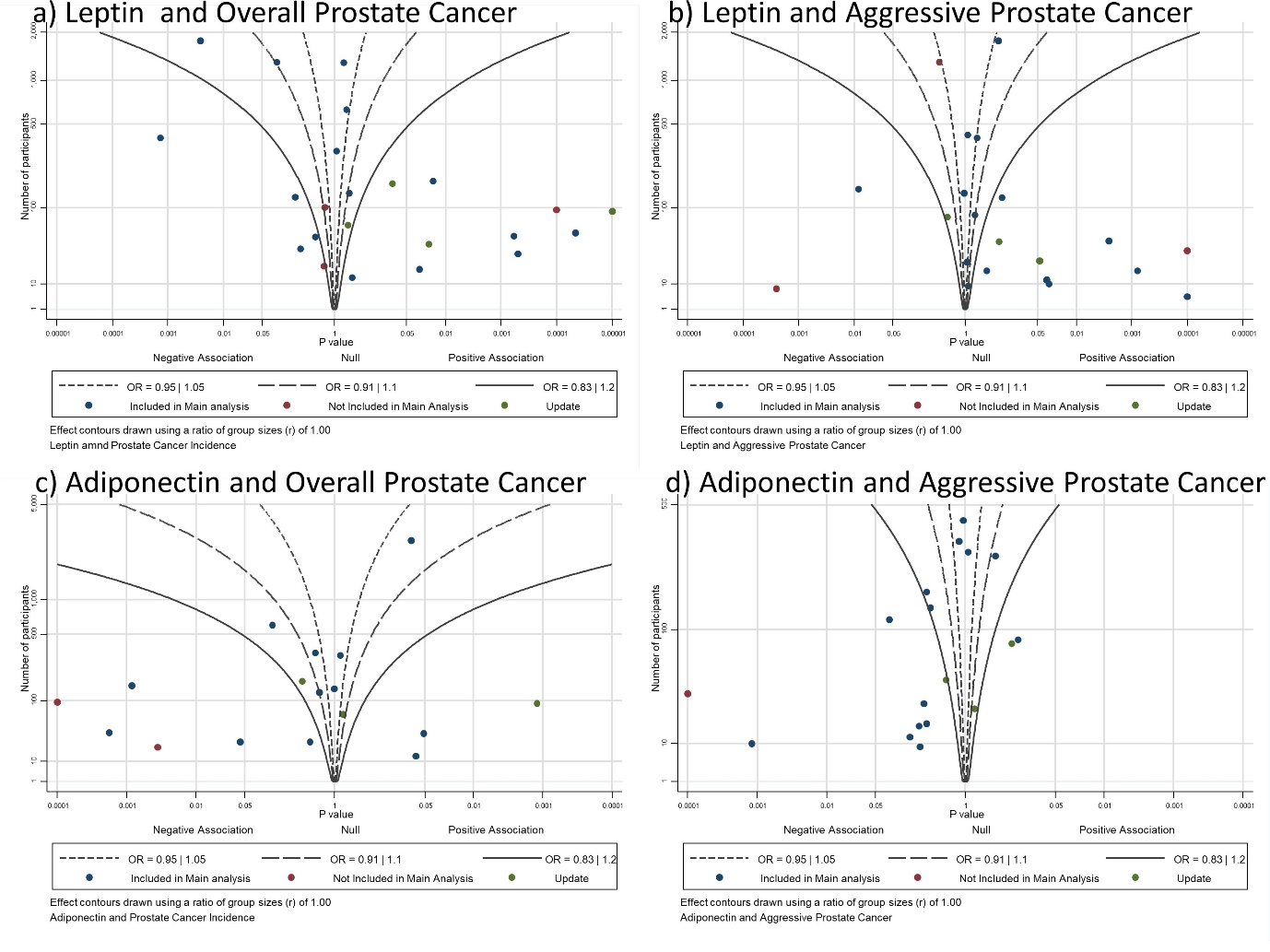

Supplement: Supplementary file 1 — Supplementary Information. [file 41598_2020_79345_MOESM1_ESM.docx]
